# Factors Affecting Long-Survival of Patients with Breast Cancer by Non-Mixture and Mixture Cure Models Using the Weibull, Log-logistic and Dagum Distributions: A Bayesian Approach

**DOI:** 10.31557/APJCP.2020.21.2.485

**Published:** 2020

**Authors:** Shideh Rafati, Mohammad Reza Baneshi, Abbas Bahrampour

**Affiliations:** 1 *Department of Biostatistics and Epidemiology, *; 2 *Modeling in Health Research Center, Institute for Futures Studies in Health, Kerman University of Medical Sciences, Kerman, Iran. *

**Keywords:** Breast cancer, survival, cure models, Bayesian

## Abstract

**Background:**

Breast cancer is a top biomedical research priority, and it is a major health problem. Therefore, the present study aimed to determine the prognostic factors of breast cancer survival using cure models.

**Methods:**

In this retrospective cohort analytic study, data of 140 breast cancer patients were collected from Ali Ibn Abi Taleb hospital, Rafsanjan, Southeastern Iran. Since in this study, a part of the population had long-term survival, cure models were used and evaluated using DIC index. The data were analyzed using Openbugs Software.

**Results:**

In this study, of 140 breast cancer patients, 23 (16.4%) cases died of breast cancer. Based on the findings, the Bayesian nonmixture cure model, with type I Dagum distribution, was the best fitted model. The variables of BMI, number of children, number of natural deliveries, tumor size, metastasis, consumption of canned food, tobacco use, and breastfeeding affected patients’ survival based on type I Dagum distribution.

**Conclusion:**

The results of the present study demonstrated that the Bayesian nonmixture cure model, with type I Dagum distribution, can be a good model to determine factors affecting the survival of patients when there is the possibility of a fraction of cure. In this study, it was found that adapting a healthy lifestyle (eg, avoiding canned foods and smoking) can improve the survival of breast cancer patients.

## Introduction

Breast cancer is the most common cancer affecting women worldwide, and its incidence and mortality rates are expected to significantly increase in the future (Anastasiadi et al., 2017). Over 1.5 million women (25% of all women with cancer) are annually diagnosed with breast cancer worldwide (Sun et al., 2017). The worldwide number of breast cancer diagnoses is expected to increase to approximately 3.2 million per year by 2030 (Winters et al., 2017).

Breast cancer is the most prevalent cancer and the second cause of cancer-related death among Iranian women, following gastric carcinoma (Asadabadi et al., 2014; Karimi et al., 2014; Moqaddasi-Amiri and Bahrampour, 2015; Rezaianzadeh et al., 2017). The mean age of Iranian breast cancer patients is 30.0 years; these patients are diagnosed with cancer at least a decade earlier compared with the developed countries(Abedi et al., 2016; Rahimzadeh et al., 2016). 

As this cancer occurs progressively in women worldwide, it is of paramount importance to identify factors that affect the survival of breast cancer patients. An assumption behind common survival models such as Cox regression model is that all patients will eventually experience the event of interest if follow-up time is sufficiently long (Swain et al., 2016). Due to recent medical advances, this assumption may not hold in diseases where there is a possibility of a fraction cure. For example, standard survival models are not usually appropriate in many childhood cancers and some adult cancers such as leukemia, colon cancer, and head-and-neck cancer, as they do not consider the possibility of cure (Kim et al., 2007). Thus, cure fraction models have been introduced.

Cure models are specific types of survival models in which the population under study is a combination of susceptible cases (those who may experience the event, namely, patients with short-term survival) and cured/non-susceptible individuals who have long-term survival (those who never experience the event in the follow-up period) (Coelho-Barros et al., 2017). In the literature, there are two major approaches (mixture and nonmixture) to model survival data with a cure fraction.

The adequacy of follow-up time, as well as the existence of a long, stable flatness with high censoring rate at the tail of the Kaplan–Meier (K-M) survival curve, indicates that the data are suitable for cure models (Othus et al., 2012). This long tail indicates that a proportion of population has a long survivorship that may correspond to cured individuals. 

To identify cured individuals, statistical tests can be used to detect patients with long-term survival. This hypothesis indicates that all people may experience death and there is no cure fraction. To accept or reject the stated hypothesis, critical values that have been set by Maller and Zhou (1996) can be used (Hoseini et al., 2017).

In this study, to overcome the drawback of small sample size, mixture and nonmixture cure models with Bayesian approach were used to identify the prognostic factors related to survival of breast cancer patients in a 15-year cohort.

Bayesian inference methods for survival data with a cure fraction were introduced by some authors, including Martinez et al., (2013); Martinez and Achcar, (2018), Swainet al., (2016), Chenet al., (1999), Ibrahim et al., (2014), and Castroet al., (2009). 

However, to date, no study has been done on Bayesian analysis of the mixture and nonmixture cure fraction models based on Dagum distribution and compared them with Weibull and Log-logistic distributions. Therefore, in this research, the mentioned models were used and compared. This may be helpful for future studies and analysis of medical and health-related data and may also help to identify the variables that affect the survival of breast cancer patients. 

## Materials and Methods

*Data*

A total of 140 breast cancer patients who referred to Ali Ibn Abitaleb hospital, Rafsanjan, Southeastern Iran, participated in this retrospective cohort study between 2000 and 2015.

Recorded data of patients were collected from noncontagious diseases software and patients’ medical records available at the Health Department of Rafsanjan University. The checklists were completed for each patient using the data collected, phone calls, and face to face interviews.

Death of patients to breast cancer was shown as failure and patients who survived until the end of the study were known as censored. The response variable was the time interval between the diagnosis, patient’s death, and the end of the study period. Independent variables, including smoking tobacco or tobacco exposure, consumption of canned food, last breastfeeding, number of children, number of caesarean sections, tumor size, stage of disease, body mass index (BMI), tumor metastasis situation, history of hypertension, history of hormonal disease, family history of malignant tumors, and family history of benign tumors, were taken into consideration.

*Statistical Analysis*

In this section, the two cure models (mixture and non-mixture) are introduced; it is shown how the Bayesian analysis of cure models is performed.

*Models*

*Mixture cure fraction models*

A mixture cure fraction model, as the name suggests, is a mixture of two types of survivals. In this model, population is divided into two parts. Cured or long term survivors and uncured or short term survivors. Let p (0 < p < 1) be the probability of being cured and so (1 - p) is the probability of an individual being susceptible (Jafari-Koshki et al., 2014). The corresponding survival function at time t is as follows: 

$S\left(t\right)=p^{F_{0}(t)}=\exp[\left(\mathrm{Inp}\right)F_{0}\left(t\right)]$

where S(t) is survival function for total population. S0(t) is the baseline survival function for the susceptible individuals (Ying et al., 2017), which in this study is assumed type I Dagum, Weibull and Log-logistic distributions for it. In the following, the mentioned distributions were introduced.

*Non-mixture cure fraction models*

In this case, the survival function is defined as

$F_{0}\left(t\right)={\left[1+\frac{1}{a}t^{-b}\right]}^{-c}={\left(\frac{1+{\mathrm{at}}^{b}}{{\mathrm{at}}^{b}}\right)}^{-c}$

Where F_0_(t) = 1 – S_0_(t) is the baseline cumulative distribution function for the susceptible individuals. 

We applied the logistic function to model the cure probability (p) under both mixture and non-mixture cure models (Martinez et al., 2013).

*The type I Dagum distribution*

Assume that survival time for the susceptible individuals has the Dagum distribution with three parameters. The cumulative distribution function of this distribution is given by (for t > 0)

$S\left(t\right)=P^{{F_{0}}^{\left(t\right)}}=\exp[\left(\mathrm{Inp}\right)F_{0}\left(t\right)]$

where b and c are positive shape parameters and a is the scale parameter (α= exp(X_i_' θ) the covariates can be included in the model through α). Note that the case c =1 leads to the log-logistic distribution.

Also, Weibull distribution with two parameters α,γ>0 (S_0_(t)=exp (-γt^α^)) which are the shape and scale parameters respectively, is assumed as the third distribution. 

*Priors*

In Bayesian analysis, the normal prior distributions N(0, 50) was considered for vector of the parameter θ. Also, the gamma prior distribution was assumed for the shape parameters in Dagum, Weibull, and log-logistic distributions. For all cases, prior independence of the parameters was assumed in the model.

*Bayesian Inference*

In this study, Bayesian analysis of the mixture and nonmixture cure fraction models was used based on Dagum, Weibull and log-logistic distributions. The joint posterior distribution for the parameters of the model was obtained by combining the joint prior distribution with the likelihood function.

Posterior summaries of interest are obtained from simulated samples for the joint posterior distribution using standard Markov Chain Monte Carlo (MCMC) procedures. Also, 1,001,000 samples were generated for each parameter of interest. The first 1000 simulated samples were discarded as a burn-in period, which is usually used to minimize the effect of the initial values. The posterior summaries of interest were based on 10,000 samples, taking every 100 sample to have approximately uncorrelated values. 

The Bayes estimates of the parameters were obtained as the mean of Gibbs samples, which were drawn from the joint posterior distribution. Convergence of the MCMC algorithm was monitored by history, autocorrelation, and quantiles plots for the simulated samples. Inferences were obtained using OpenBUGS Software.

*Model Selection*

Comparison between mixture and non-mixture models assuming different distributions was assessed using the Deviance Information Criteria (DIC) as a measure of the goodness-of-fit, where a lower DIC value indicates better model fit. 

## Results

The study was conducted on 140 patients with breast cancer in a 96 months period (15 years and 6 months). Of total patients, 23 (16.4%) cases faced the event of death due to breast cancer and 117 (83.6%) cases are censored. The 5, 10 and 15 years survival rate of patients were about 0.82, 0.71 and 0.71, respectively. Table 1 has described demographic, clinical, and laboratory characteristics of patients.

**Figure 1:**
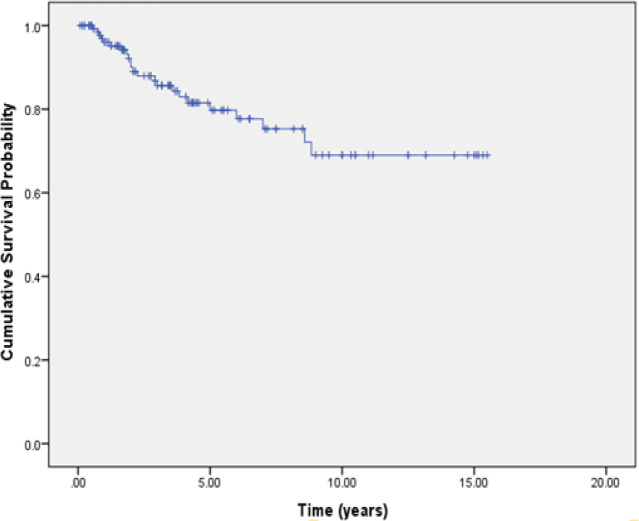
Kaplan–Meier Estimate of the Overall Survival Function for the Breast Cancer Data

Figure 1 displays a Kaplan -Meier plot for overall survival function, which shows “flatness” near 0.65 in the survival curve, and thus a cure fraction model appears to be suitable for this data. Based on this diagram, flatness occurs after about 10 years and the curve stabilized for about five years. 

In Table 2, the DIC values of Bayesian cure models based on type I Dagum, Log-logistic and Weibull distributions and in the presence all of covariates have been reported. 

Based on Table 2, the DIC value of log-logistic mixture cure model (320.2) is less than the non-mixture cure model (322.9); however, the difference of DIC value for two models is less than 5, so the performance of these two models does not have difference significant (Lin, 2014). 

Also it has been indicated that Non-mixture type I Dagum is better than the others and Weibull models (mixture and non-mixture) are the worst. Therefore, in the following (table 3), the results of Bayesian non-mixture cure model under type I Dagum distribution were presented.

The posterior summaries of parameters of non-mixture cure models in the presence of covariates and under type I Dagum distribution have been presented in Table 3. 

In section short term survival in Table 3, the 95% credible interval for BMI, number of children, natural delivery, tumor size and metastasis does not include zero. Therefore, these variables have a significant effect on the short term survival. 

Based on this table, by controlling other factors, the odds of failure (death) for patients who have BMI more or equal 25 is 0.21 less than others (OR=exp(-0.242)). 

For one unit of increase in the number of children, the failure odds is reduced by 0.16 (OR=exp(-0.170)). The death odds of patients who have metastasis is 0.10 more than others (OR=exp(0.097)). Findings showed that for one unit of increase in the number of natural delivery and tumor size, the odds of death is increased by 0.04 and 0.11, respectively.

Also, in section long term survival, the 95% credible interval for consumption of canned food, Tobacco use, tumor size and the duration of last breastfeeding does not include zero suggesting that these variables have significant effect on the long term survival. 

This Table showed that the cure odds of patients who consume canned food is 0.36 less than others (OR=exp(-0.455)). Moreover, by controlling other factors, the cure odds of patients who use tobacco is roughly 0.17 less than others (OR=exp(-0.186)). Increasing one unit in tumor size decrease the cure odds by 0.35 (OR=exp(-0.431)). Findings revealed that for one unit of increase in the duration of breastfeeding, the cure odds is reduced 0.19 by adjusting the effect of other variables (OR=exp(-0.214)). Based on the results in Table 3, it is shown that tumor size is significant in two parts (short-term and long-term survival) but the other variables as mentioned above are effective only in long-term or short-term survival.

**Table 1:** Patients Characteristics

Variables	Censored (n=117)		Died (n=23)	
Continuous variables	Mean	SD	Mean	SD
Diagnosis age of cancer	46.65	10.21	49.39	20.31
Number of children	3.85	2.29	5.22	3.53
Tumor size	14.22	9.33	33.78	43.46
Duration of the last lactation (month)	15.97	9.04	16	6.29
Categorical variables	Number	Percent	Number	Percent
BMI (Less than 24.99)	39	33.3	12	52.2
BMI (More or equal to 25)	78	66.7	11	47.8
Stage of disease (II)	91	77.8	3	13
Stage of disease (III)	24	20.5	11	47.8
Stage of disease (IV)	2	1.7	9	39.1
Tobacco use	40	34.2	13	56.5
Tumor metastasis	60	51.3	23	65.7
History of hypertension	61	52.1	14	60.9
History of hormonal disease	18	15.4	4	17.4
History of breast benign tumor	15	12.8	5	21.7
Family history of benign tumor	23	19.7	2	8.7
Family history of malignant tumor	56	47.9	12	52.2

**Table 2:** DIC Values of Bayesian Cure Models

Models	DIC
Mixture type I Dagum	315.6
Non-mixture type I Dagum	194
Mixture Log-logistic	320.2
Non-mixture Log-logistic	322.9
Mixture Weibull	421.3
Non-mixture Weibull	489.6

**Table 3:** Posterior Summaries of the Type I Dagum Cure Models

Parameter	Posterior mean	Posterior SD**	95%Credible Interval	Odds Ratio
Long-term survival				
Consumption of canned food	-0.455	0.137	(-0.693,-0.273)*	0.634
Tobacco use	-0.186	0.133	(-0.327,-0.008)*	0.83
Tumor size	-0.431	0.143	(-0.599,-0.222)*	0.65
The duration of last breastfeeding (month)	-0.214	0.1	(-0.392,-0.099)*	0.807
Short-term survival				
BMI	-0.242	0.053	(-0.298,-0.143)*	0.785
Number of children	-0.17	0.027	(-0.197,-0.127)*	0.844
natural delivery	0.04	0.017	(0.011,0.061)*	1.04
Tumor size	0.103	0.003	(0.004,0.150)*	1.11
metastasis	0.097	0.048	(0.017,0.171)*	1.101

*, significant at 95% level; **, Standard Deviation

## Discussion

The aim of this study was to assess and compare Bayesian mixture and nonmixture cure fraction models based on the Weibull, Log-logistic, and Dagum distributions and determine factors affecting the survival of breast cancer patients.

The Swain study showed the utility of generalized Gompertz distribution under the mixture and nonmixture cure models based on Bayesian approach. Their research findings showed that the mixture cure model has a better fit than the nonmixture cure model (Swain et al., 2016).

A study was conducted to compare mixture and non-mixture cure fraction models based on the generalized modified Weibull distribution under Bayesian approach. The DIC values for the two models (mixture and non-mixture) also provided very close results (Martinez et al., 2013). Another study aimed to review mixture and nonmixture cure models and found that both classes fit the data well (Othus et al., 2012).

Inferences for the mixture and the nonmixture cure models under the Bayesian approach using the Weibull distribution showed that the nonmixture model was better fitted by the data (Achcar et al., 2012). No similar study has determined the survival of breast cancer patients using cure models under type I Dagum distribution.

In the present study, based on DIC values of the models, Bayesian nonmixture cure model under type I Dagum distribution was found to be the best fitted model. The significance of the variables of BMI, number of children, natural delivery, tumor size, metastasis, consumption of canned food, tobacco use, and breastfeeding was confirmed based on type I Dagum distribution.

In the previous studies, which aimed to determine the risk factors associated with breast cancer, the Bayesian mixture cure fraction model was used, based on Generalized Modified Weibull distribution. In those studies, as in the present study, tumor metastasis status was found to be an effective factor in the survival of patients (Karimi et al., 2014; Naseri et al., 2018).

Based on the findings of the present study, tobacco use was a factor influencing the survival of these patients. This outcome agreed with that of the previous studies (Macacu et al., 2015; Andersen et al., 2017; Sun et al., 2017; Winters et al., 2017). 

There is evidence to suggest that smaller family size and less breastfeeding decrease the survival of breast cancer patients (Winters et al., 2017). The first finding was in accordance with the present study, but the second finding was not. However, similar to the present study, Triver’s study confirmed that shorter duration of breastfeeding was associated with a decreased risk of death (Trivers et al., 2007).

Overweight and obese premenopausal women (40-49 years) had, respectively, 14% and 26% lower risk of developing breast cancer compared to premenopausal women with normal weight (Winters et al., 2017). Some studies have shown a lower survival rate in underweight women or in those women who experience unexplained weight loss after diagnosis (McTiernan et al., 2010). This outcome agrees with the present study that revealed the odds of death for patients who had a BMI of higher or equal to 25 was less than others. In other words, higher BMI had a protective effect.

Poor dietary habits appear to be linked to increased risk of death in breast cancer patients. Based on the results of this study, women who consumed canned food had a lower survival rate compared to those women who did not. Perhaps, this finding can be justified by the fact that canned foods contain a lot of nitrate and nitrate is considered a cause of cancer. This result has been confirmed in previous studies that indicated nutrition is an important factor in the survival of breast cancer patients (Winters et al., 2017; Seiler et al., 2018). 

The results of this study have shown that one of the most important factors affecting the survival of breast cancer patients is tumor size. This finding has been confirmed by previous studies, as they revealed that patients with smaller tumor size survived longer (Abedi et al., 2016; Nikbakht and Bahrampour, 2017; Sarveazad et al., 2018). 

To date, no study has assessed the performance of type I Dagum distribution and compared it with log-logistic and Weibull distributions to identify the variables that affect the survival of breast cancer patients in both general classes of cure fraction model under Bayesian approach, which was done in this study for the first time. However, This study had some limitations. Many factors are associated with the survival of breast cancer patients, including type of treatment, physical activity, modern lifestyles (excessive alcohol consumption and too much fat intake), and early menarche/late menopause, which were not assessed in this study. Thus, it is suggested to conduct other studies with more effective factors to determine the survival of patients with breast cancer.

As conclusion, the results of the current study demonstrated that the Bayesian nonmixture cure model under type I Dagum distribution can be a good selection to determine factors affecting the survival of breast cancer patients where there is the possibility of a fraction of cure. In this study, it was found that adapting a healthy lifestyle (eg, avoiding canned foods and smoking) can improve the survival of breast cancer patients.
